# Physical Activity and Survival among Long-term Cancer Survivor and Non-Cancer Cohorts

**DOI:** 10.3389/fpubh.2017.00019

**Published:** 2017-02-14

**Authors:** Anthony S. Gunnell, Sarah Joyce, Stephania Tomlin, Dennis R. Taaffe, Prue Cormie, Robert U. Newton, David Joseph, Nigel Spry, Kristjana Einarsdóttir, Daniel A. Galvão

**Affiliations:** ^1^Exercise Medicine Research Institute, Edith Cowan University, Joondalup, WA, Australia; ^2^School of Population Health, University of Western Australia, Nedlands, WA, Australia; ^3^Public Health and Clinical Services Division, Western Australian Department of Health, East Perth, WA, Australia; ^4^School of Medical and Health Sciences, Edith Cowan University, Joondalup, WA, Australia; ^5^School of Human Movement and Nutrition Sciences, The University of Queensland, St. Lucia, QLD, Australia; ^6^Institute for Health and Ageing, Australian Catholic University, Melbourne, VIC, Australia; ^7^University of Queensland Centre for Clinical Research, The University of Queensland, Brisbane, QLD, Australia; ^8^Department of Radiation Oncology, Sir Charles Gairdner Hospital, Nedlands, WA, Australia; ^9^Faculty of Medicine, University of Western Australia, Nedlands, WA, Australia; ^10^Genesis Cancer Care, Joondalup, WA, Australia; ^11^Centre of Public Health Sciences and Unit for Nutrition Research, School of Health Sciences, University of Iceland, Reykjavik, Iceland

**Keywords:** physical activity, cancer, survival, longitudinal, cohort study

## Abstract

Evidence suggests physical activity improves prognosis following cancer diagnosis; however, evidence regarding prognosis in long-term survivors of cancer is scarce. We assessed physical activity in 1,589 cancer survivors at an average 8.8 years following their initial diagnosis and calculated their future mortality risk following physical activity assessment. We also selected a cancer-free cohort of 3,145 age, sex, and survey year group-matched cancer-free individuals from the same source population for comparison purposes. Risks for cancer-specific mortality and all-cause mortality in relation to physical activity levels were estimated using Cox regression proportional hazard regression analyses within the cancer and non-cancer cohorts. Physical activity levels of 360+ min per week were inversely associated with cancer-specific mortality in long-term cancer survivors [hazard ratios (HR) = 0.30 (95% confidence intervals (CI) 0.13–0.70)] and participants without prior cancer [HR = 0.16 (95% CI 0.05–0.56)] compared with no reported physical activity. Physical activity levels of 150–359 and 360+ min were inversely associated with all-cause mortality in long-term cancer survivors [150–359 min; HR = 0.55 (95% CI 0.31–0.97), 360+ min; HR = 0.41 (95% CI 0.21–0.79)] and those without prior cancer [150–359 min; HR = 0.52 (95% CI 0.32–0.86), 360+ min; HR = 0.50 (95% CI 0.29–0.88)]. These results suggest that meeting exercise guidelines of 150 min of physical activity per week were associated with reduced all-cause mortality in both long-term cancer surviving and cancer-free cohorts. Exceeding exercise oncology guidelines (360+ min per week) may provide additional protection in terms of cancer-specific death.

## Introduction

Identification and management of lifestyle risk factors affecting prognosis in cancer survivors is becoming increasingly important as cancer screening and treatments continue to improve. In Australia, the number of cancers diagnosed almost doubled between 1991 and 2009, with a corresponding increase in age-standardized incidence of 12% (1). During a similar period of time, 5-year relative survival following any cancer diagnosis increased from 47% in 1982–1987 to 66% in 2006–2010 (1).

Assessment of physical activity levels and their effects in those who survived cancer has been undertaken by a number of researchers (2, 3); however, the usual period of assessment has been within a relatively short period following cancer diagnosis (almost exclusively less than 2 years). These studies have generally shown positive associations between increasing levels of physical activity and improved quality of life, cancer-specific mortality, and all-cause mortality for survivors of certain cancer types, particularly breast (4), colorectal (5–7), and prostate (8) cancer. Additionally, it has been suggested that cancer recurrence might be positively impacted by physical activity levels post-diagnosis, although evidence appears contradictory (9–12). To date, however, assessment of physical activity levels in terms of their effects on mortality in long-term survivors has been almost non-existent, although a recent study by Inoue-Choi et al. (13) suggested improved survival benefits for cancer survivors may be associated with adherence to the recommended physical activity guidelines. With improved long-term survival in those diagnosed with cancer, it is important to understand whether the survival benefits associated with physical activity extend beyond the immediate rehabilitation stage associated with cancer (and treatment) recovery. Moreover, it is important to assess whether physical activity behaviors contribute similar benefits in these long-term cancer survivors, compared to cancer-naive individuals. By linking the Western Australian Cancer Registry dataset with the Western Australian Health and Wellbeing Surveillance System (HWSS) dataset, we were able to obtain self-reported leisure-time physical activity (LTPA) levels at time points between 2 and 28 years following first recorded cancer diagnosis. This allowed us to investigate whether long-term survivors of “any cancer” benefited from increased physical activity, in terms of future cancer-specific and all-cause mortality risk.

## Materials and Methods

### Ethics Statement

The study was approved by the Human Research Ethics Committees of Edith Cowan University and the Western Australian Department of Health and has therefore been performed in accordance with the ethical standards laid down in the 1964 Declaration of Helsinki and its later amendments.

### Study Design

This population-based cohort study utilized self-reported lifestyle survey information (from the HWSS) individually linked with cancer registry data (both held by the Western Australian Department of Health). The HWSS is a comprehensive monthly survey commissioned by the Western Australian Department of Health to provide information on a wide range of issues pertaining to the Western Australian population’s physical and mental well-being. It utilizes computer-assisted telephone interviewing to assess approximately 6,000 Western Australians each year who are selected from the WA White Pages^®^ telephone directory using a stratified random process with over-sampling representative to the population in rural and remote areas. Each year since its inception, more than 75% of those contacted completed the survey (14) and a majority (77% in 2010) of participants provided their name, address, and date of birth for the purpose of linkage with administrative health data. Only those HWSS participants who provided consent for their information to be used in this manner were linked to other registries for this study. The probabilistic matching procedures used to link individuals are based on full name and address, phonetic compression algorithms, and other identifiers, and they have been estimated to be 99.89% accurate (15). This linkage allowed identification of incident cancer diagnoses prior to an initial survey date and provided information on behavioral factors and demographics at the time of survey. Mortality data were obtained through linking to the Western Australian Mortality Registry for the entire study period (2004–2011).

### Study Population

Between May 1, 2004 and January 1, 2011, some 44,317 surveys for which consent was provided for data linkage were completed as part of the HWSS. Where participants had been surveyed more than once (1,616 people) during the study period, their last survey was included for analysis. Upon further restriction to those aged 40+ years, to target adult cancers only, 25,433 participants remained. Those with a diagnosed cancer (other than non-melanoma skin cancer) after 1982 and more than 2 years prior to their survey date were identified (1,813). Their first cancer within this period was considered their incident cancer. After further exclusion of cancer survivors with multiple cancers diagnosed prior to the survey, a cohort of 1,667 cancer survivors was selected. Exclusion of a further 78 individuals with missing information for body mass index (BMI), alcohol consumption, and/or SF-8 questions (Table 1) left a cancer survivor cohort of 1,589. These participants were surveyed on average 8.8 years (quartile 1 = 4 years, quartile 3 = 12 years) following their first recorded cancer diagnosis.

**Table 1:** **Cancer survivor cohort: baseline characteristics by level of leisure-time physical activity (LTPA), in participants surveyed an average of 8.8 years following initial reported cancer diagnosis**.

	No LTPA (*n* = 439)	<150 min LTPA (*n* = 460)	150–359 min LTPA (*n* = 384)	360+ min LTPA (*n* = 384)
	
	*n* (%)	*n* (%)	*n* (%)	*n* (%)
Male	205 (46.7)	187 (40.6)	173 (45.0)	184 (47.9)

Age at survey (years)*				
40–49	18 (4.1)	28 (6.1)	26 (6.8)	35 (9.1)
50–59	58 (13.2)	86 (18.7)	75 (19.5)	76 (25.8)
60–69	111 (25.3)	133 (28.9)	122 (31.8)	137 (27.2)
70–79	146 (33.3)	138 (30.0)	114 (29.7)	102 (26.6)
80+	106 (24.2)	75 (16.3)	47 (12.2)	34 (8.8)

Body mass index (kg/m^2^)*				
<25	131 (29.8)	168 (36.5)	137 (35.7)	134 (34.9)
25–29	157 (35.8)	155 (33.7)	159 (41.4)	162 (42.2)
30+	119 (27.1)	120 (26.1)	74 (19.3)	76 (19.8)
Missing	32 (7.3)	17 (3.7)	14 (3.6)	12 (3.1)

Daily fruit and/or vegetable intake*				
<4 servings	129 (29.4)	106 (23.0)	87 (22.7)	63 (16.4)
4–5 servings	172 (39.2)	191 (41.5)	126 (32.8)	142 (37.0)
6 servings	64 (14.6)	84 (18.3)	69 (18.0)	63 (16.4)
7+ servings	74 (16.9)	79 (17.2)	102 (26.6)	116 (30.2)

Smoking status				
Never	187 (42.6)	220 (47.8)	188 (49.0)	176 (45.8)
Ex-smoker	202 (46.0)	194 (42.2)	161 (41.9)	181 (47.1)
Current	50 (11.4)	46 (10.0)	35 (9.1)	27 (7.0)

Alcohol consumption (# drinks on any given day)*				
None	165 (37.6)	169 (36.7)	104 (27.1)	95 (24.7)
≤2 standard drinks	199 (45.3)	223 (48.5)	197 (51.3)	197 (51.3)
>2 standard drinks	75 (17.1)	67 (14.6)	83 (21.6)	92 (24.0)
Missing	0	1 (0.2)	0	0

SF-8 physical health component score*				
PCS < 50	300 (68.3)	267 (58.0)	180 (46.9)	127 (33.1)
PCS ≥ 50	137 (31.2)	193 (42.0)	204 (53.1)	256 (66.7)
Missing	2 (0.5)	0	0	1 (0.3)

SF-8 mental health component score*				
MCS < 54	231 (52.6)	247 (53.7)	181 (47.1)	154 (40.1)
MCS ≥ 54	206 (46.9)	213 (46.3)	203 (52.9)	229 (59.6)
Missing	2 (0.5)	0	0	1 (0.3)
Diabetes mellitus (% yes)*	77 (17.5)	62 (13.5)	34 (8.8)	24 (6.2)

Previous cancer type				
Breast	98 (22.3)	107 (23.3)	88 (22.9)	79 (20.6)
Prostate	75 (17.1)	78 (17.0)	57 (14.8)	70 (18.2)
Colorectal	50 (11.4)	56 (12.2)	55 (14.3)	43 (11.2)
Melanoma	76 (17.3)	84 (18.3)	70 (18.2)	91 (23.7)
Other	140 (33.9)	135 (29.4)	114 (29.7)	101 (26.3)
Years from diagnosis to survey [mean (Q1–Q3)] (mean = 8.82; 4–12)	9.2 (5–13)	8.7 (4–12)	8.6 (4–12)	8.7 (4–13)

*Significantly associated with LTPA (*p* < 0.05).

A non-cancer cohort (NCC) was selected from the same source population of 44,317 individuals. Stratified random sampling without replacement at a ratio of 2:1 was performed using the age (10-year age blocks from 40 years onward), sex, and survey year frequency distributions of the 1,667 cancer survivors identified previously. This was performed using the “proc surveyselect” function within SAS Inc. software (version 9.3). The resulting 3,334 individuals had no cancer diagnosis prior to their survey (Table 2). After exclusion of 189 individuals with missing data for BMI and SF-8 questions, a final 3,145 cancer-free individuals were included in the final analyses.

**Table 2:** **Non-Cancer cohort: baseline characteristics by level of leisure-time physical activity (LTPA) in survey participants without a prior recorded cancer diagnosis**.

	No LTPA (*n* = 886)	<150 min LTPA (*n* = 882)	150–359 min LTPA (*n* = 801)	360+ min LTPA (*n* = 765)
	
	*n* (%)	*n* (%)	*n* (%)	*n* (%)
Male*	407 (45.9)	353 (40.0)	339 (42.3)	399 (52.2)

Age at survey (years)*				
40–49	42 (4.7)	49 (5.6)	55 (6.9)	68 (8.9)
50–59	108 (12.2)	149 (16.9)	165 (20.6)	168 (22.0)
60–69	239 (27.0)	232 (26.3)	267 (33.3)	268 (35.0)
70–79	286 (32.3)	284 (32.2)	225 (28.1)	205 (26.8)
80+	211 (23.8)	168 (19.0)	89 (11.1)	56 (7.3)

Body mass index (kg/m^2^)*				
<25	290 (32.7)	315 (35.7)	359 (44.8)	336 (43.9)
25–29	315 (35.6)	303 (34.4)	284 (35.5)	306 (40.0)
30+	197 (22.2)	208 (23.6)	132 (16.5)	102 (13.3)
Missing	84 (9.5)	56 (6.4)	26 (3.2)	21 (2.8)

Daily fruit and/or vegetable intake*				
<4 servings	276 (31.2)	234 (26.5)	167 (20.8)	146 (19.1)
4–5 servings	323 (36.5)	336 (38.1)	283 (35.3)	276 (36.1)
6 servings	125 (14.1)	157 (17.8)	152 (19.0)	129 (16.9)
7+ servings	162 (18.3)	155 (17.6)	199 (24.8)	214 (28.0)

Smoking status*				
Never	393 (44.4)	479 (54.3)	411 (51.3)	370 (48.4)
Ex-smoker	349 (39.4)	307 (34.8)	330 (41.2)	338 (44.2)
Current	144 (16.2)	96 (10.9)	60 (7.5)	57 (7.4)

Alcohol consumption (# drinks on any given day)*				
None	335 (37.8)	316 (35.8)	220 (27.5)	170 (22.2)
≤2 standard drinks	417 (47.1)	441 (50.0)	441 (55.1)	415 (54.2)
>2 standard drinks	134 (15.1)	125 (14.2)	140 (17.5)	180 (23.5)
Missing	0	0	0	0

SF-8 physical health component score*				
PCS < 50	563 (63.5)	446 (50.6)	305 (38.1)	224 (29.3)
PCS ≥ 50	322 (36.3)	436 (49.4)	495 (61.8)	541 (70.7)
Missing	1 (0.1)	0	1 (0.1)	0

SF-8 mental health component score*				
MCS < 54	443 (50.0)	421 (47.7)	338 (42.2)	293 (38.3)
MCS ≥ 54	442 (49.9)	461 (52.3)	462 (57.7)	472 (61.7)
Missing	1 (0.1)	0	1 (0.1)	0
Diabetes mellitus (% yes)*	129 (14.6)	120 (13.6)	80 (10.0)	62 (8.1)

*Significantly associated with LTPA (*p* < 0.05).

### Study Variables

Participants were asked to estimate the number of sessions and minutes per session of LTPA during the past week, in terms of walking 10 or more minutes consecutively, performing moderate physical activity (e.g., golf, gentle swimming, and lawn bowls), or vigorous physical activity (e.g., tennis, jogging, and cycling). Using recommendations of the Australian Institute of Health and Welfare (16), total LTPA was calculated using the formula [total LTPA = walk-time + moderate-time + (2 × vigorous-time)]. Sufficient LTPA was defined as follows: no LTPA, <150 min LTPA, 150–359 min LTPA, or 360+ min LTPA per week. While Australian physical activity guidelines (16) collapse the upper two categories (150–359 min and 360+ min), given the approximately equal number of participants in the two upper categories, stratification was preferable in this instance. For those aged 65+ years, no weighting was applied for vigorous physical activity in order to improve comparability between years due to the question not being asked for that age group prior to 2008. Few respondents aged 65+ years reported being vigorously active from 2008 onward. For those whose total LTPA per week exceeded 1,680 min, their summed value was re-coded to 1,680 min for analysis as recommended in the Australian Health and Welfare guidelines (16).

Confounding variables included in the adjusted Cox regression analyses were sex, age at survey, previous cancer type (none, breast, prostate, colorectal, melanoma, other), smoking status (never more than 100 cigarettes, ex-smoker, current-smoker), fruit and/or vegetable intake (<4, 4–5, 6, 7+ servings daily; based on quintile distribution), BMI (<25, 25–29, 30+ kg/m^2^; adapted from World Health Organization classifications), long-term risky alcohol intake (none, ≤2 standard drinks, 3+ standard drinks on a drinking day; based on National Health and Medical Research Council guidelines), SF-8 physical health component score (<50, 50+; based on median values), SF-8 mental health component score (<54, 54+; based on median values), year of survey, and self-reported diabetes status. The SF-8 Health Survey component of the HWSS is an eight item version of the SF-36^®^. Higher SF-8 scores correspond to better functioning.

Two outcomes were investigated following the participants’ surveys, namely cancer-specific mortality and all-cause mortality. Cancer death was identified using the International Classification of Diseases version 10 codes C00-D48, present as either principal or other cause of death.

### Statistical Analyses

Differences in baseline characteristics in relation to level of LTPA were assessed using the Kruskall–Wallis (for ordinal variables) and chi square (for nominal variables) tests.

Person-time was calculated from the date of survey until death, or end of follow-up (January 1, 2011), whichever occurred first. Cox proportional hazards regression models were used to estimate hazard ratios (HR) with 95% confidence intervals (CI) for future mortality. Separate analyses were performed for the two outcome types (cancer-specific and all-cause mortality), with non-cancer mortality being censored in cancer-related analyses.

The final adjusted Cox models, which incorporated a stratum of “time from cancer diagnosis until survey” for the cancer survivor cohort, included LTPA, previous cancer type, age, sex, smoking status, BMI, daily fruit and vegetable intake, survey year, long-term risky alcohol use, SF-8 physical and mental health component scores, and self-reported diabetes. Socioeconomic index for areas and region (metro or regional residence) were not included in the adjusted model as they were not significantly associated with the outcome in crude Cox models.

Differences in survival as a function of prior cancer status (yes/no) were assessed by combining the CCs and NCCs, and introducing a variable denoting prior cancer status to the adjusted Cox regression model. All aforementioned covariates were also included in this model.

Trends for the effects of LTPA on the two outcomes were estimated by excluding the categorical LTPA variable from the class statement in the adjusted Cox regression model. To test for interaction between LTPA and prior cancer status, the two cohorts were combined, and interaction terms [LTPA × prior cancer status (yes/no)] were added to the models.

The proportional hazards assumption that the ratio of mortality rates according to the exposure variable remained constant for the adjusted models was tested by inclusion of an interaction term between the LTPA variable and log(survival time). No violation of the proportional hazards was observed.

For all analyses, a two sided *p* < 0.05 was considered statistically significant. The statistical software SAS 9.3 was used to perform all analyses.

## Results

### Baseline Characteristics

At time of survey, the median age for both the CC (*n* = 1,589) and the NCC (*n* = 3,145) was 68 years [interquartile range (IQR): 60–76 years]. Median BMI was similar between cohorts (CC = 26.6 kg/m^2^, IQR = 23.8–29.9; NCC = 26.0 kg/m^2^, IQR = 23.4–29.2), as was median fruit/vegetable servings per day (CC = 5.0 servings, IQR = 4.0–6.0; NCC = 5.0 servings, IQR = 4.0–6.0), median SF-8 physical health component score (CC = 49.6, IQR = 39.9–54.4; NCC = 51.1, IQR = 42.3–55.6), median SF-8 mental health component score (CC = 54.1, IQR = 48.1–57.7; NCC = 54.8, IQR = 49.3–57.7), and percentage with self-reported diabetes (CC = 11.8%; NCC = 11.5%). Percentage of current smokers (CC = 9.5%; NCC = 10.5%) and long-term risky drinking (>2 standard drinks on any given day) (CC = 19.8%; NCC = 18.0%) varied slightly between cohorts.

### LTPA-Stratified Baseline Characteristics in the CC

Compared with those who reported no LTPA per week, those reporting increased levels of LTPA tended to be younger, have lower BMI, greater fruit/vegetable and alcohol intake, and were less likely to be current smokers (Table 1). Mental health component scores from SF-8 questions were appreciably higher in the 360+ min of LTPA group and an apparent dose-response between increasing levels of LTPA and percentage of those with SF-8 physical health component scores above the median was observed (Table 1). All of the aforementioned factors were significantly associated with LTPA aside from gender and smoking status. In terms of time from first cancer diagnosis until survey, a slightly higher mean number of years were observed in the “no LTPA” group compared to the other LTPA levels. However, no significant association between LTPA and “years from cancer diagnosis until survey” was observed.

### LTPA-Stratified Baseline Characteristics in the NCC

The relationships between LTPA-stratified baseline characteristics for the NCC (Table 2) appeared to mirror those observed in the CC; however, significant associations between LTPA and gender, and LTPA and smoking status were observed in the NCC that were not present within the CC. This may relate in part to the greater number of individuals present in the NCC.

### Cohort Follow-up

The median duration of follow-up after survey was 2.6 years (out to 7.6 years) for the cancer survivor cohort, during which time 83 cancer-specific deaths and 135 all-cause deaths occurred. In comparison, a median duration of 2.8 years (out to 7.6 years) for the NCC was observed, during which time 52 cancer-specific deaths and 152 all-cause deaths occurred.

### Survival in Relation to Prior Cancer Status

Prior cancer was associated with a threefold increased risk [HR = 3.05 (95% CI 2.15–4.33)] for cancer-specific mortality, compared to those without prior cancer after adjustment for LTPA, age, sex, smoking status, BMI, daily fruit and vegetable intake, survey year, long-term risky alcohol use, SF-8 physical and mental health component scores, and self-reported diabetes. Adjusted estimates of risk for all-cause mortality were 72% higher [HR = 1.72 (95% CI 1.36–2.17)] in those with a prior reported cancer, compared to those with no prior cancer reported.

### LTPA and Cancer-Specific Mortality

Risks for cancer-specific mortality in participants with prior cancer [HR = 0.30 (95% CI 0.13–0.70)] and without prior cancer [HR = 0.16 (95% CI 0.05–0.56)] were significantly reduced in those reporting 360 min or more of LTPA per week, compared to those reporting none (Table 3). For both prior-CCs and NCCs there appeared to be an inverse dose–response relationship between level of LTPA and risk of cancer-specific mortality (CC *p*_trend_ = 0.0024; NCC *p*_trend_ = 0.0016). However, no significant interaction was observed between prior cancer status and LTPA in relation to cancer-specific mortality (*p*_interaction_ = 0.8341) risk.

**Table 3:** **Risk for cancer-specific death and all-cause death by weekly leisure-time physical activity (LTPA) levels, in cohorts with and without previously reported cancer diagnosis**.

		Outcomes following physical activity assessment					
		Cancer-specific death			All-cause death		
	At risk (*n*)	No. of events	Hazards ratio (HR)^a^	95% confidence intervals (CI)	No. of events	HR^a^	95% CI
LTPA (minutes per week)							
Cancer cohort (CC)							
None	405	40	Ref.	Ref.	67	Ref.	Ref.
<150	443	24	0.62	0.36–1.06	38	0.70	0.46–1.08
150–359	370	12	0.55	0.28–1.08	18	0.55	0.31–0.97
360+	371	7	0.30	0.13–0.70	12	0.41	0.21–0.79
Total participants	1,589	83			135		

Non-cancer cohort							
None	801	26	Ref.	Ref.	73	Ref.	Ref.
<150	826	13	0.66	0.33–1.31	41	0.78	0.52–1.15
150–359	774	10	0.54	0.25–1.15	21	0.52	0.32–0.86
360+	693	3	0.16	0.05–0.56	17	0.50	0.29–0.88
Total participants	3,145	52			152		

*^a^Cox model includes age at survey, sex, smoking category, long-term risky drinking category, body mass index category, daily fruit and vegetable intake, survey year, self-reported diabetes, SF-8 mental health component score, SF-8 physical health component score, and previous cancer type (for CC)*.

### LTPA and All-Cause Mortality

All-cause mortality during the follow-up period was significantly reduced by 45–59% (Table 3) for those reporting 150–359 or 360+ min per week of LTPA, regardless of prior cancer status. While not significant, results also suggested some reduction in risk for those performing less than 150 min LTPA per week. Significant trends were observed in terms of effects from increasing levels of LTPA on reduction in all-cause mortality, for the cancer (*p* = 0.0178) and non-cancer (*p* = 0.0215) cohorts. No significant interaction was observed between prior cancer status and LTPA in relation to all-cause mortality (*p*_interaction_ = 0.9932) risk.

## Discussion

This observational study aimed to investigate the relationship between LTPA and cancer-specific mortality, and between LTPA and all-cause mortality, in two cohorts; a long-term cancer survivor cohort and a cohort without prior recorded cancer who were frequency matched on age, gender, and survey year to those in the CC. Results confirmed an association between 150 min or more LTPA and reduced all-cause mortality in both cohorts. In relation to cancer-specific mortality, physical activity exceeding 360 min per week was associated with survival benefits regardless of a person’s prior cancer status. Lower levels of physical activity (<150, 150–359 min per week) were not significantly associated with reductions in cancer-specific mortality in those with or without a prior cancer.

Physical activity has previously been shown to provide immediate beneficial effects for cancer survivors, including improvements in physiological markers, body composition, physical function, fatigue, and psychological outcomes (2, 17–20). Although evidence related to long-term cancer survivors is sparse, a recent study by Inoue-Choi et al. (13) suggested adherence to recommended levels of physical activity in long-term cancer survivors may improve all-cause, CVD-specific, and cancer-specific mortality. While the study variables and design used by Inoue-Choi et al. differed to those in our study in that we included men and women, a comparative non-cancer group, and different doses of physical activity (e.g., 150, 150–359, and 360+ min per week), it was of interest to note the clear protective (adjusted) effects of physical activity that existed for both cancer-specific and all-cause mortality outcomes in their study. Similarly, a recent prospective cohort study (21) of 830 long-term prostate cancer survivors assessed physical activity at 2.5, 4.7, and 6.8 years post-diagnosis (comparable to the 8.8-year assessment time point in our study) showed a protective effect of increased physical activity and prostate cancer mortality. In our study, the likelihood of cancer-related death for cancer survivors appeared to decrease for increasing levels of physical activity culminating in a significant 70% reduced risk for those performing 360+ min of LTPA per week. In the same exposure group (360+ min), all-cause mortality in cancer survivors was reduced by almost 60%.

In our cohort containing individuals with and without prior cancer, we observed a 72% increased all-cause mortality risk over the follow-up period in cancer survivors compared to those without a prior cancer. We also observed a threefold increased risk for cancer-specific mortality in those with prior cancer compared to cancer-naive participants. Higher risk of non-cancer-related mortality may in part be explained by an overrepresentation of CVD risk factors being observed in cancer survivors (22). Although it would have been interesting to test for CVD-specific mortality, we did not investigate this due to the relatively low numbers of cancer survivors having a CVD-specific death recorded and the likelihood of competing risks between the CVD and cancer mortality outcomes.

For long-term cancer survivors, previous evidence suggests an increased risk for both cancer and non-cancer-related mortality compared to that of the general population (23, 24). A number of studies have highlighted the above average incidence of second primary or recurrent cancers in those surviving an initial cancer (25). This increased risk for subsequent cancer appears to depend upon cancer type and/or cancer treatment, and other individual-specific risk profiles (e.g., lifestyle, genetics, and other exposures) (25). In addition, increases in all-cause and non-cancer-related mortality in cancer survivors have been reported (13). There are a number of mechanisms by which physical activity may further improve cancer-specific survival in long-term cancer survivors as well as those without prior cancer. Since both cohorts (those with and those without prior cancer) observed apparent cancer-specific survival benefits from physical activity, one explanation might be that physical activity reduces the likelihood of cancer incidence, resulting in fewer cancer deaths. However, in some unpublished analyses from this study we observed no significant relationship between physical activity and cancer incidence (or second primary cancer incidence in the prior-CC). An alternative explanation is that physical activity may play a role in improving prognosis of those who develop an incident or second primary cancer. Evidence supporting this has been reported by a number of researchers (26). Although the biological mechanisms through which this is achieved are still unclear, there are a number of promising avenues. The influence of exercise on host factors such as metabolic hormones, inflammation/cytokines, and immune surveillance have been suggested, as have exercise’s effects on certain tumor-related factors such as p27, CTNNB1, CACNA2D3, and L3MBTL1 (26).

By selecting an NCC from the same source cohort who possessed similar age and gender distributions as the cancer survivors, we investigated whether differential physical activity-related effects on our two outcomes (cancer mortality and all-cause mortality) might exist. Certainly, physical activity has been associated with reduced all-cause mortality rates (27) and cardiovascular-related disease (28–30) or cardiovascular-death (31) in population-based cohorts. Results from our study of a positive association between physical activity and decreasing cancer-specific and all-cause mortality in our NCC reflected previous findings. Moreover, our results suggested the benefits of moderate to high levels of physical activity in decreasing cancer-specific and all-cause mortality were comparable between those with or without a prior cancer. From a health promotion and management perspective, this provides a degree of confirmation that physical activity recommendations for the general public are applicable and beneficial for cancer survivors. Moreover, these benefits were observed in an aggregate cancer survivor group and, while cancer type was accounted for in the analyses, it is likely these benefits of physical activity would apply broadly to survivors of most cancer types.

There are a number of strengths attributable to this study. Foremost was our ability to gain all recorded retrospective and post-survey cancer and mortality records for those who participated in the survey. This allowed an assessment of physical activity levels an average 8.8 years following cancer survivors’ initial cancer diagnoses. Second, the in-depth survey of participants allowed adjustment of numerous confounding variables associated with lifestyle, physical, and mental health, which provides a greater reliability in estimating the association between physical activity and the outcomes of interest. In addition, access to non-cancer survey participants (at time of survey) enabled comparison of physical activity influences on the outcomes in participants with and without a prior cancer. It is unlikely there would be many instances of misclassification of cancer outcomes as these were mostly derived from pathology laboratories or radiation oncologists (32). For similar reasons, mortality records are unlikely to be a basis for misclassification bias.

Some limitations exist with this study. Given the self-reported nature of the physical activity measurements, some misclassification of the exposure might exist. Any associated bias is likely reduced by the use of quite broad categories for physical activity (0, <150, 150–59, 360+ min per week) and is unlikely to relate to the outcomes due to the prospective nature of the study. While it made sense to classify physical activity based on recommendations and health promotion messages, our aggregation of low, moderate, and vigorous physical activity in calculating amount of LTPA per week excluded our ability to measure differences in effectiveness between low, moderate, and vigorous levels of physical activity. Moreover, although we adjusted for a number of potential confounders in our analyses, possible confounding may still exist and contribute to the identified associations. In addition, loss to follow-up (between survey and outcome) would likely have been minimal since the individuals were followed up through the Western Australian Health registries—in terms of cancer and/or death. This means that virtually all deaths and cancers reported throughout the follow-up period would be included; however, those occurring outside of Western Australia would presumably be lost to follow-up. Finally, the observational nature of the study does not permit us to infer cause and effect.

In summary, this study suggests physical activity is associated with improved cancer-specific and all-cause survival in long-term survivors of cancer. These associations were comparable in magnitude to those seen in a NCC of similar age and gender, selected from the same source population. Evidence also suggested further benefits in survival may be achieved by those exceeding 360 min of LTPA per week, regardless of an individual’s prior cancer status.

## Author Contributions

AG, SJ, ST, DT, PC, RN, DJ, NS, KE, and DG: substantial contributions to the conception or design of the work or the acquisition, analysis, or interpretation of data for the work; drafting the work or revising it critically for important intellectual content; final approval of the version to be published; agreement to be accountable for all aspects of the work in ensuring that questions related to the accuracy or integrity of any part of the work are appropriately investigated and resolved.

## Conflict of Interest Statement

The authors declare that the research was conducted in the absence of any commercial or financial relationships that could be construed as a potential conflict of interest. The handling Editor declared a shared affiliation, though no other collaboration, with several of the authors (SJ and ST) and states that the process nevertheless met the standards of a fair and objective review.
